# Gut microbiota composition and frailty in elderly patients with Chronic Kidney Disease

**DOI:** 10.1371/journal.pone.0228530

**Published:** 2020-04-01

**Authors:** Elisabetta Margiotta, Francesco Miragoli, Maria Luisa Callegari, Simone Vettoretti, Lara Caldiroli, Maria Meneghini, Francesca Zanoni, Piergiorgio Messa

**Affiliations:** 1 Division of Nephrology, Dialysis and Renal Transplantation, Fondazione IRCCS Cà Granda Ospedale Maggiore Policlinico, Milano, Italy; 2 Centro di Ricerche Biotecnologiche, Università Cattolica del Sacro Cuore, Cremona, Italy; 3 Università degli Studi di Milano, Milano, Italy; Weill Cornell Medicine, UNITED STATES

## Abstract

**Background:**

Frailty is common in older patients affected by chronic kidney disease (CKD). Since gut microbiota (gMB) may contribute to frailty, we explored possible associations between gMB and frailty in CKD.

**Methods:**

We studied 64 CKD patients (stage 3b-4), categorized as frail (F, 38) and not frail (NF, 26) according to Fried criteria, and 15 controls (C), all older than 65 years. In CKD we assessed serum C-reactive protein, blood neutrophil/lymphocyte ratio, Malnutrition-inflammation Score (MIS); gMB was studied by denaturing gel gradient electrophoresis (DGGE), high-throughput sequencing (16S r-RNA gene), and quantitative real-time PCR (RT-PCR).

**Results:**

No differences in alpha diversity between CKD and C and between F and NF patients emerged, but high-throughput sequencing showed significantly higher abundance of potentially noxious bacteria (*Citrobacter*, *Coprobacillus*, etc) and lower abundance of saccharolytic and butyrate-producing bacteria (*Prevotella* spp., *Faecalibacterium prausnitzii*, *Roseburia* spp.), in CKD respect to C. *Mogibacteriaceae* family and *Oscillospira* genus abundance was positively related to inflammatory indices in the whole CKD cohort, while that of *Akkermansia*, *Ruminococcus* and *Eubacterium* genera was negatively related. Compared with NF, in F there was a higher abundance of some bacteria (Mogibacteriacee, Coriobacteriacee, *Eggerthella*, etc), many of which have been described as more abundant in other diseases.

**Conclusions:**

These results suggest that inflammation and frailty could be associated to gMB modifications in CKD.

## Introduction

Frailty is highly frequent in patients affected by chronic kidney disease (CKD) (14–68%), its prevalence increases with age and progression of kidney disease, and it has been associated with worse quality of life and poor outcomes [1]. The ERA-EDTA guidelines suggest to evaluate the presence of frailty in older patients with advanced CKD in order to better stratify their overall risk and to program targeted rehabilitative interventions [2]. Growing evidence accumulated in the last decade suggests that gut microbiota (gMB) composition may play a causal role in determining the inflammatory and oxidative stress status in many clinical settings [3,4].

gMB changes have been described both in frail subjects and in CKD patients, with potential worsening of their clinical outcomes [5,6].

As a matter of fact, gut dysbiosis has been reported in frail patients, and it is characterized by a significant reduction in the number of *Lactobacillus*, *Faecalibacterium prausnitzii and Bacteroides-Prevotella* groups, with a concomitant increase of Enterobacteriaceae [6]. Changes of gMB composition, with increased Enterobacteria and reduced Lactobacillaceae and Prevotellaceae, have been reported also in CKD, but only in few studies mainly focused on end stage renal disease (ESRD) [7,8]. The coexistence of CKD-specific pathological conditions (dietary restrictions, drugs, sedentary lifestyle, low fluid intake, slowed intestinal transit time, comorbidities), can enhance the potential proinflammatory effects of gMB changes, leading to increased risk of inflammation, malnutrition and, eventually, global frailty [9–11].

Furthermore, aging is associated with increased chronic inflammation related to sarcopenia. Sarcopenia is also typical of CKD patients, as a consequence of reduced physical activity and increased adiposity, and it induces low-grade chronic inflammation, the so called Inflammaging. Inflammaging is emerging as a central pathologic mechanism of aging, which predisposes to frailty and age-associated chronic diseases [12].

The prevalence of elderly and frail CKD patients is progressively increasing. Examining the relationship between CKD and gMB in these patients, might give new insights for improving clinical management of this high-risk cohort. Most of the few studies previously published on this topic were carried out only in dialysis patients.

Therefore, the aim of this study is to explore the prevalence of frailty in a cohort of older pre-dialysis CKD patients, in relation to gMB composition, and to examine possible gMB differences between frail and not frail CKD patients.

## Methods

### Study design

In this observational study we evaluated cross sectionally 64 CKD patients (eGFR<45 ml/min/1.73m2 not on dialysis), aged ≥ 65 years, enrolled from a cohort of 101 prevalent CKD patients in continuous follow-up at the outpatient clinic of the Department of Nephrology of Policlinico Ospedale Maggiore of Milan.

The study protocol was reviewed and approved by Ethics Committee of Comitato Etico Milano Area 2; a written informed consent was signed by all participants. Partecipants were recruited from 1st September 2015 to 6th December 2016. All eligible patients that fulfilled the inclusion criteria were screened during the observational period and were asked to participate to the study. 37 patients were excluded according to the exclusion criteria.

Exclusion criteria were inflammatory and/or autoimmune diseases and/or ongoing immunosuppressive treatment for these pathologies (i.e. calcineurin inhibitors, steroids, methotrexate, mycophenolic acid), cancer, use of probiotics/antibiotics within 3 months before study entry, and inability to collaborate.

CKD patients were compared with 15 healthy controls (C) with normal renal function (eGFR >60ml/min/1.73m^2^) that were recruited among the relatives and friends of the researchers involved in the project. Control subjects were matched for age and had to fulfill all the selection criteria that were applied to CKD patients except of renal impairment. All eligible CKD patients were classified into frail (F-CKD) and not frail (NF-CKD) according to Fried’s Frailty Phenotype (FFP) score. Frail patients had to fulfil 3 of the following 5 criteria: a) weight loss, b) walking slowness, c) exhaustion, d) weakness, e) low physical activity[13]. eGFR was calculated from standard creatinine (determined by colorimetric method) using the CKD-EPI equation [14].

Nutritional assessment was evaluated through: serum albumin, serum transferrin, body mass index (BMI), and the Malnutrition Inflammation Score (MIS) questionnaire, which consists of ten components, each of them envisaging 4 levels of severity, from 0 (normal) to 3 (severely malnourished), with a total score ranging from 0 to 30 [15]. The evaluation of body composition was assessed by multiphase Bioelectrical Impedance Analysis, BIA (Body Composition Monitor, Fresenius Medical Care, Bad Homburg, Germany). We evaluated also some inflammatory indices, such as serum C-reactive protein (CRP, dosed by turbidimetric method) and blood neutrophil to lymphocyte (N/L) ratio, that were correlated with gMB composition. Nutritional and inflammatory parameters have been assessed only in CKD population.

Individuals were requested to complete a questionnaire regarding antibiotics, probiotics and/or any immunosuppressive drugs used within the month before fecal sample collection. Furthermore, all subjects were asked to fill a diary regarding dietary intake in the two days before the stool collection (these data were subsequently elaborated with Winfood software, Medimatica Surl). Feces were collected by each volunteer at home on the day preceding the visit, using 20 ml plastic sterile stool collection containers and placed in their own freezer at -18/-20°C overnight, before being stored at -80°C in our laboratories until analysis.

### DNA extraction and PCR-DGGE

Bacterial DNAs were extracted from 50 mg of fecal sample using the FastDNA^™^ SPIN Kit for Soil (MP Biomedicals, Switzerland) following the manufacturer’s instructions. DNA was eluted with 100 μl of elution buffer and stored at −20°C until further analysis. Then extracted DNAs were used as target in PCR reactions with universal primers (Hda1GC-Hda2) targeting the 16SrRNA gene [16]. The amplification conditions, gel composition and run have been already described by Miragoli et al. 2016 [17]. Gel were analyzed with Fingerprinting II SW software (Bio-Rad Laboratories, Hercules, CA, USA) using Pearson’s coefficient, using the Unweighted Pair Group Method with Arithmetic Mean algorithm (UPGMA). The Shannon Wiener Diversity (H) Index of the biodiversity was calculated using the previously reported formula.[18]

As DGGE can only provide a qualitative estimate of the levels of a specific bacterial group, we subsequently performed Illumina sequencing of V3-V4 regions of 16S rRNA gene.

### High-throughput sequencing and bioinformatic analysis

PCR amplifications were carried out using the primers 343F (5’TACGGRAGGCAGCAG 3’) and 802R (5’TACNVGGGTWTCTAATCC 3’). A specific tag (7 nucleotides) was attached to forward primer for demultiplex of sequences during bioinformatics analysis. The PCR amplification was performed in triplicate using 1 ng of DNA for each reaction. The PCR protocol included an initial denaturation (95°C, 3 min), followed by 25 cycles of denaturation at 94°C for 30 sec, annealing at 52°C for 30 sec and extension at 72°C for 30 sec and elongation at 72°C for 7 min. Each reaction was carried out in a 25 μl of mixture containing 1 μl of DNA, 0.5 μM of each primer. To check eventual contaminations during PCR reaction assembly, each reaction mixture was prepared in duplicate. One reaction was prepared as already mentioned the other one represented the negative control to which 1 μl of water was added. The PCR products were checked by agarose gel electrophoresis and then quantified using the Qubit HS dsDNA fluorescence assay (Life Technologies, Carlsbad, CA, USA). Amplicons were polled in equimolar concentration (20 ng each) and then purified by the Agencourt AMPure XP PCR1 Purification system (Beckman Coulter, Brea, CA, USA).

Sequencing was performed using Illumina’s MiSeq platform (Parco Tecnologico Padano, Lodi, Italy) with 300 bp paired-end mode and v3 chemistry. After demultiplexing and quality check the reads were trimmed using Qiime 1.9. This allows the reads to be truncated after base quality dropping below 15 (Phred-scale) and reads ID to be compatible for the following QIIME pipeline scripts. We used the 97% clustered Qiime formatted Greengenes v.13.8 reference database. Alpha diversity index metrics (observed OTUs, Chao1, Observed species, Shannon, Simpson and Goods coverage) were calculated using Qiime pipeline. PCA was performed on OTUs using post hoc test Tukey-Kramer by Stamp software. The linear discriminant analysis (LDA) with effect size measurements (LEfSe) was used to identify indicator bacterial groups within the microbial communities.

### Real time PCR (RT-PCR)

To confirm statistical analysis RT-PCR reactions were performed to quantify representative group of bacteria resulted statistically significant. The RT-PCR reactions were performed for *Eggerthella lenta*, *Lactobacillus* spp. *F*. *prausnitzii*, and *Citrobacter* spp. as already described by Cho et al [19], Byun et al [20], Sokol et al [21] and Patel et al [22], respectively. Moreover, *Roseburia* spp. and *Prevotella*-*Bacteroides* group quantification was performed as described by Larsen et al [23]. Standard curves were prepared by decimal dilution of reference DNAs. The RT-PCR reactions were performed in a LightCycler® 480 Instrument II (Roche Life Science, Mannheim, Germany) and amplification conditions were set up in order to achieve optimal reaction efficiency. Results were expressed as percentage of ng of specific target gene/ ng of total DNA.

### Statistical analysis

Values for results were expressed as means ± SD or medians ±IQR. Continuous variables were compared using the unpaired t-test and categorical variables were compared using the Chi-squared analysis. When data were non-normally distributed, two-tailed nonparametric Mann-Whitney test was used instead. Canonical correspondence analysis (CCA) was performed using Pearson correlation coefficient to test the association between discriminant group of bacteria and clinical parameters across the three groups of individuals using the R package microbiome. The Benjamini and Hochberg’s FDR-controlling procedure was used for multiple comparisons.

Statistical significance was determined using two-tailed Student’s t test to compare samples and one or two-way ANOVA to compare samples from different groups of individuals. GraphPad Prism 6 software was used to analyze and plot the data. P < 0.05 was considered statistically significant.

## Results

### Patients and controls

Clinical characteristics of CKD and C cohorts are shown in Table 1. In addition to the expected differences in renal function parameters, age and BMI were significantly higher in CKD patients, with a greater prevalence of female gender and diabetes compared to C.

**Table 1:** Clinical parameters among CKD patients and controls.

Characteristics	CKD (n = 64)	Controls (n = 15)	p value	
Age (years)	80.7±6.2	73.7±7.6	0.0003	*
males/females	43 /21	4/11	0.007	*
eGFR (ml/min/1,73 ^m2^)	26±11	75±11	<0.0001	*
Diabetics(n)	37	4	0.043	*
BMI (kg/m^2^)	28.4±4.7	25.5±2.9	0.0301	*

CKD, Chronic kidney disease; eGFR, estimated glomerular filtration rate; BMI, Body mass index

38 (59%) of CKD patients were categorized as frail according to the FFP scale criteria, while the remaining 26 (41%) were not frail (Table 2). Twenty-four (63%) F-CKD patients were affected by diabetes, compared to 13 (50%) in NF-CKD group, but this difference was not statistically significant. No significant differences between F- and NF-CKD groups were found in any of the explored variables, except for MIS, which was higher in F-CKD compared to NF-CKD (Table 2).

**Table 2:** Clinical parameters among F-CKD and NF-CKD patients.

Characteristics	Frail (38)	Not Frail (26)	p value	
Age (years)	81,8±5,8	79,03±6,6	0.079	
Sex, male (female)	22 (16)	21 (5)	0.55	
sCreatinine	2.36	2.65	0.24	
GFR (CKD-EPI)	25,68±9,7	26,2±13,3	0.79	
CKD IIIb patients	12	9	0.79	
CKD IV patients	26	17	0.79	
Diabetes	24	13	0.29	
BMI	28,77±5,4	27,7±3,4	0.38	
LTM (lean tissue mass) %	45,7±12,4	52,2±8,7	0.05	
CRP	0,37±0,6	0,26±0,24	0.4	
MIS	7,6±5,4	3,96±1,9	0.0017	*
Pre-albumin	27,3±4,4	29,4±6,2	0.11	
Hypertension	35	23	0.623	
CV events	22	15	0.868	
N/L	2,53±1	2,51±1	0.94	

eGFR, estimated glomerular filtration rate; CRP, C-reactive protein; BMI, Body mass index; LTM, lean tissue mass; MIS, malnutrition inflammation score, N/L neutrophil/lymphocytes ratio

### PCR-DGGE analyses

**Fig 1:**
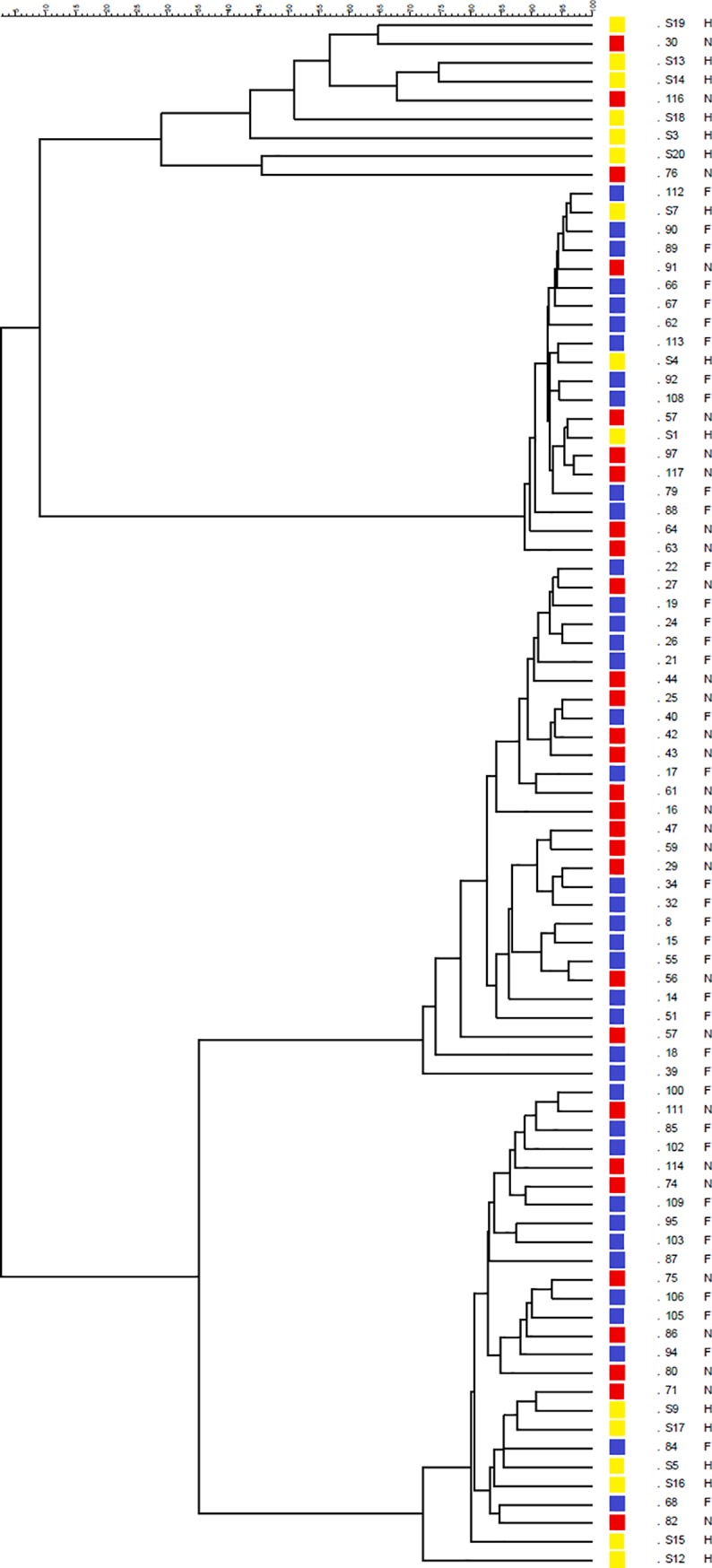
Dendrogram constructed from analysis of PCR-DGGE gels using Pearson's correlation coefficient and the unweighted-pair group method. F-CKD (blue squares); NF-CKD (red squares); C (yellow squares).

A preliminary investigation was conducted on fecal samples by mean of PCR-DGGE analysis. Software analysis of the PCR-DGGE profiles (Fig 1) provided a dendrogram in which the pattern of bands were grouped on the basis of their similarity level that graphically was indicated by the similarity coefficient; the height of the columns was inversely proportional to the similarity between the bands. The software grouped all the analyzed profiles in four clusters. F-CKD and NF-CKD patients were equally distributed in the four clusters, whereas C were grouped mainly in the first and last clusters. The first cluster grouped six out of 15 C subjects and two out of 29 NF-CKD profiles with a degree of similarity lower (from 45 to 75%) than those of the other three clusters. However, the average number of bands visible in the PCR-DGGE profiles of each group of subjects (CKD 25.2± 4.8; C 26.46 ± 2.47, F-CKD 25.26 ± 4.19; NF-CKD 25.15 ± 5.7; mean ± sd) was very similar with no statistically significant difference in biodiversity being observed.

### High-throughput sequencing in CKD compared to C

The PCR-DGGE analysis is a rapid method to detect differences in dominant microbiota composition between samples. It is a qualitative analysis based on profile comparison of amplicons obtained by amplification of 16SrRNA gene. This technique has some limitations, and in particular, allows the detection of dominant bacteria present in analyzed samples while those present in lower abundance are neglected. In order to deeply investigate the gut microbiota composition of CKD and C patients we decide to use a metabarcoding approach.

**Fig 2:**
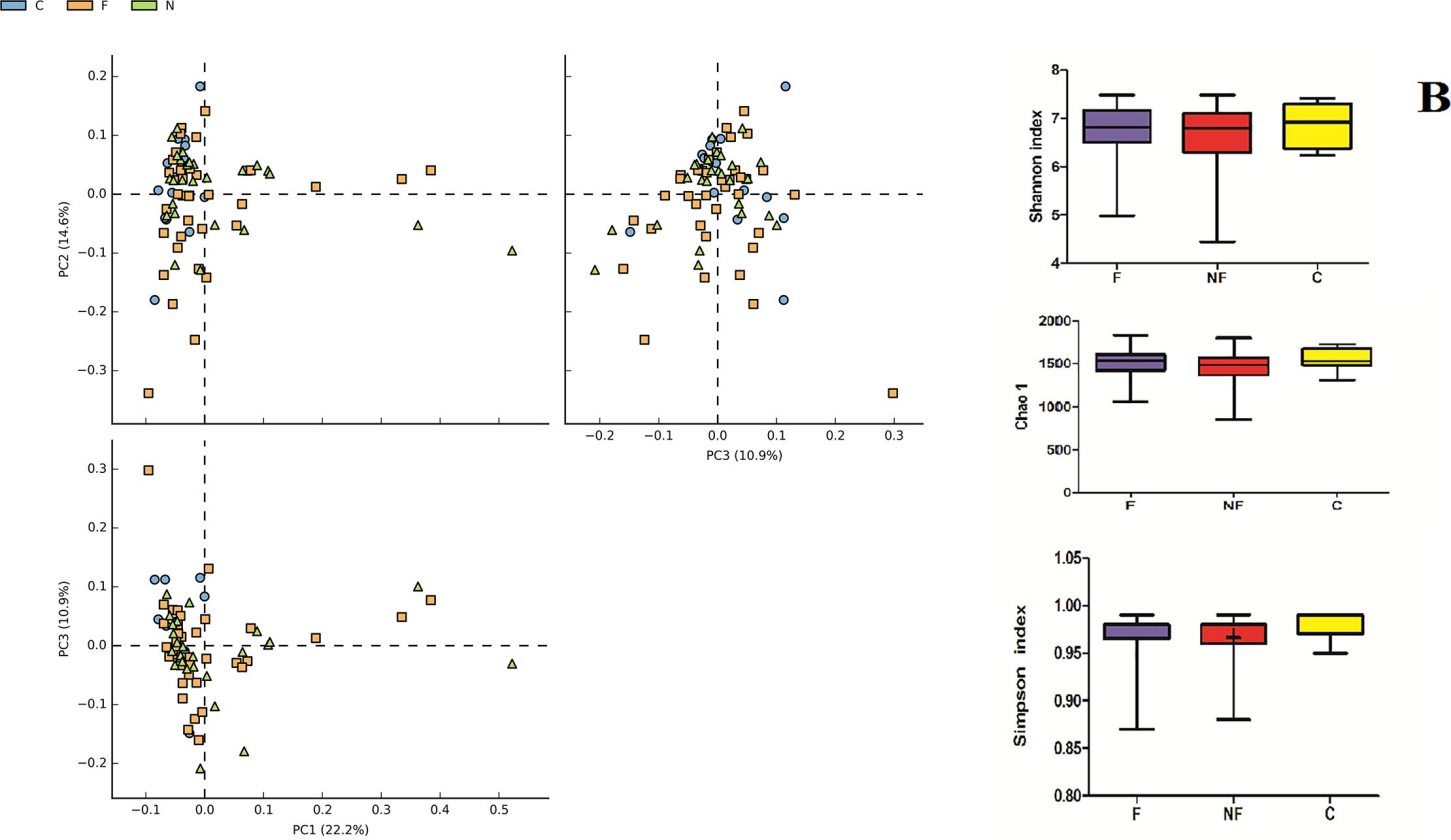
**A.** Graphical PCA of CKD-F (orange squares), CKD-NF (green triangles) and C (blue balls) microbial communities. The analysis was carried out at OTU level. **B.** Graphic summary of alpha-diversity indices. Comparison of Chao1, Shannon, and Simpson indices of gut microbiota between CKD-F, NF and Controls.

Illumina sequencing was performed on V3-V4 regions of 16S rRNA gene and OTU obtained by bioinformatics analysis were investigate by mean of alpha and beta diversity. Alpha diversity analysis revealed no significant differences in either OTU richness (Chao1 index) or OTU diversity (Simpson, Shannon index) between the three groups (F-CKD, NF-CKD and C) (Fig 2B). To find differences in fecal bacterial communities between the three groups of individuals, a principal component analysis (PCA) was performed on OTUs. PCA ordination plot did not revealed distinct clusters, suggesting that no significant differences in microbial composition were found between F-CKD, NF-CKD and C. (Fig 2A).

We also compared gMB composition of CKD- diabetic and non-diabetic patients using PCA analysis and Anova test in order to detect differential groups of bacteria. No statistically significant differences between the analyzed groups (CKD-D, CKD-ND and C) were found.

**Fig 3:**
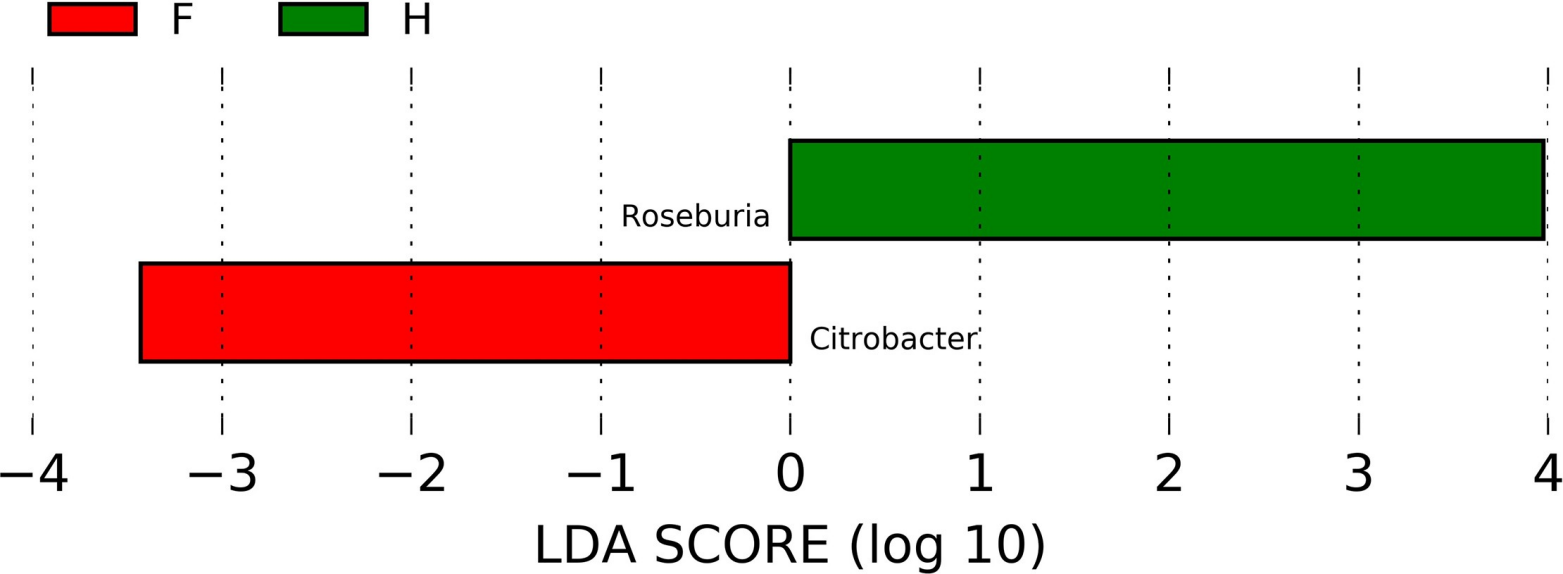
Graphical results obtained with logarithmic linear discriminant analysis (LDA) score higher than 2 determined by effect size (LefSe). Indicator bacterial groups within the CKD and Control groups were: *Citrobacter* genus resulted to be more abundant in CKD patients than in C whereas *Roseburia* spp. was higher in C than in CKD group.

**Fig 4:**
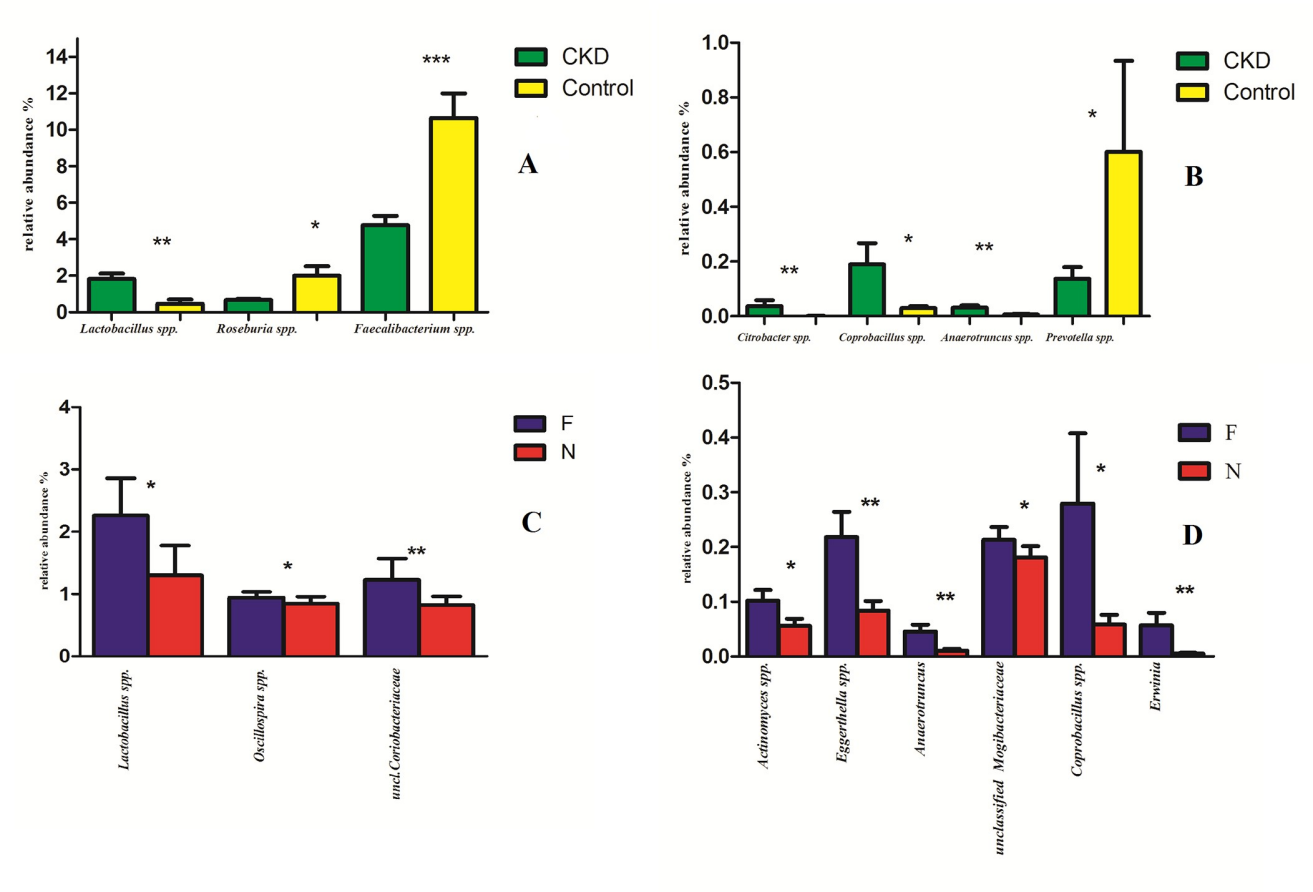
Statistically significant bacterial groups expressed as relative abundance (%). *p < 0.05, **p < 0.01. (**A, B**): Green and yellow bars represent CKD patients and healthy controls respectively. On the left, there are the bacterial genera with higher relative abundance (A), on the right those with lower relative abundance (B). (**C, D**): Blue and red bars represent Frail (F) and Not frail (N) patients respectively; bacterial genera with higher relative abundance (C), bacterial genera with lower relative abundance (D).

**Fig 5:**
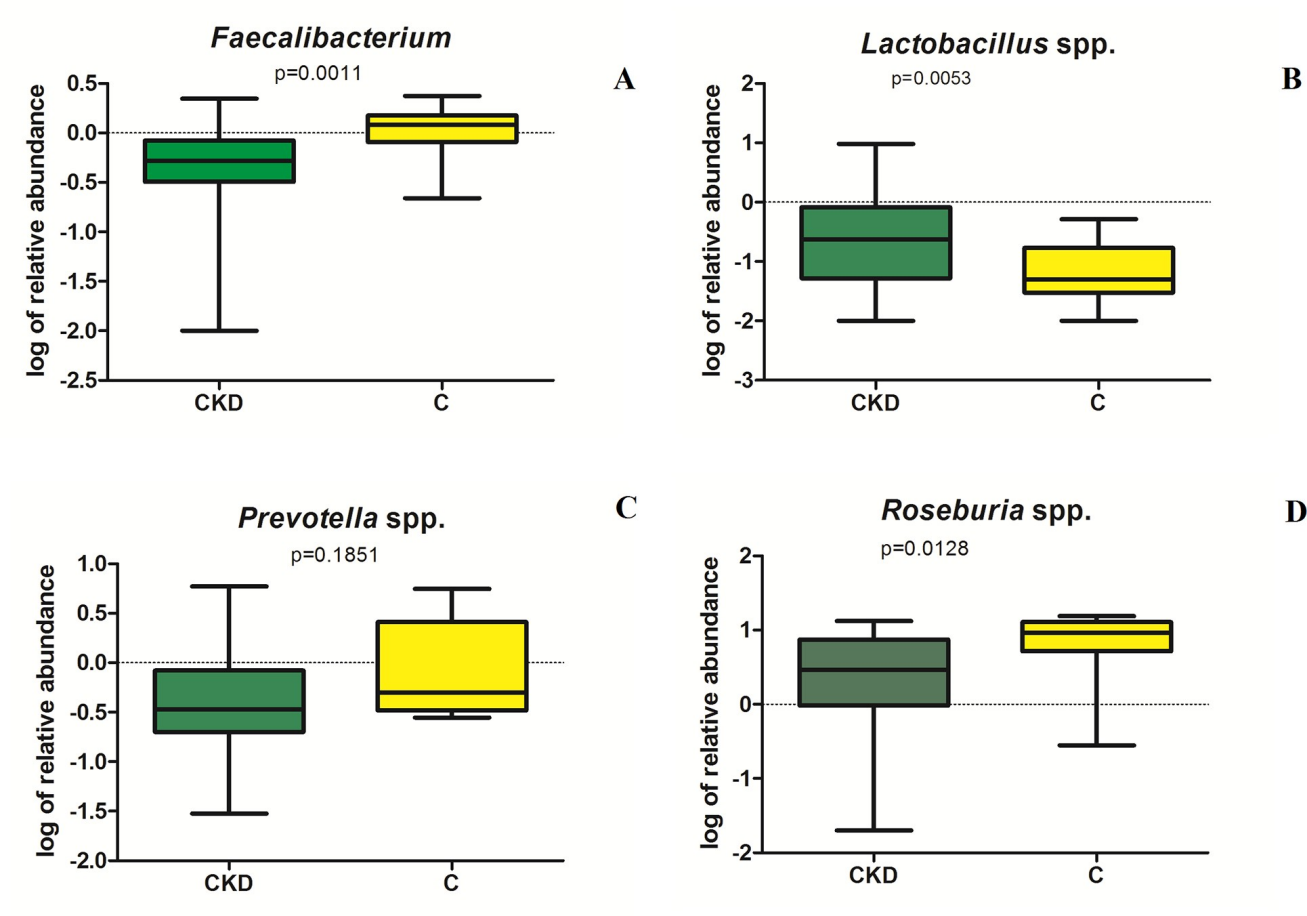
Representation of RT-PCR quantification (expressed as log of relative abundance) of species resulted to be statistically different by sequence analysis.

In order to evaluate which group of bacteria were enriched in the different groups according to metabarcoding data, linear discriminant analysis (LDA) coupled with effect size measurements (LEfSe) was applied. Lefse analysis identified *Citrobacter* spp. and *Roseburia* spp., respectively, as the most differentially abundant genera in CKD and C (Fig 3). Lower abundance of *Roseburia* spp. in CKD respect to C was confirmed also by RT-PCR (Fig 5D); unfortunately, no specific couple of primers was available for RT-PCR quantification of *Citrobacter* spp. No significant differences were found between the two groups at the family level (S1 Fig). The relative abundance of genus showed statistically significant differences between CKD and C (Fig 4A and 4B). Namely, a higher relative abundance of genera *Lactobacillus*, *Coprobacillus*, *Anaerotruncus* and *Citrobacter* and *Ruminococcus torques* species (S2 Fig) was observed. Moreover, a reduction of *Prevotella* spp., *F*. *prausnitzii* and *Roseburia* spp. (all saccharolytic and butyrate producing bacteria) were observed in CKD patients respect to controls.

RT-PCR analysis confirmed significant lower abundance of *F*. *prausnitzii* and *Roseburia* spp (p = 0.0011 and p = 0.00128 respectively, see Fig 5A and 5D) and higher *Lactobacillus* spp counts (p = 0.0053) in CKD patients compared to C (Fig 5B). No significant difference in *Prevotella* spp. content was observed in CKD and C groups (p = 0.185) (Fig 5C).

### High-throughput sequencing in F versus NF CKD patients

A statistically significant increase in the relative abundance of genera *Lactobacillus* and *Oscillospira* and Coriobacteriacee family in stools of F-CKD as compared to NF-CKD patients was observed (Fig 4C). However, the quantification of *Lactobacillus* spp. by RT-PCR did not confirm the significant differences between F and NF observed in the sequences analyses (p = 0.1958).

Furthermore, we observed a statistically significant increased abundance of *Eggerthella* (particularly *E*. *lenta* species, S2 Fig), *Erwinia*, *Anaerotruncus*, *Coprobacillus* genera and Mogibacteriacee family (Fig 4D). Moreover, an increase of OTU related to *Eubacterium dolichum*, *Eubacterium cylindroides* and *Dorea* spp in the frail group was observed (S2 Fig).

### Correlation analysis of clinical/ inflammatory indices and gMB

In CKD cohort, a positive correlation between unclassified Mogibacteriaceae and *Oscillospira* abundance with CRP and MIS levels, respectively was found, whereas *Akkermansia*, *Ruminococcus* and *Eubacterium* genera were negatively correlated to N/L ratio (Table 3).

**Table 3:** Correlation analysis of CRP, MIS and N/L values and gMB.

		*Eubacterium* spp.	*Ruminococcus* spp.	*Akkermansia* spp.	Unclass.Mogibacteriaceae Family	*Oscillospira* spp.
CRP	p	0.64	0.38	0.73	**0.029**	0.53
	R	-0.06	-0.11	-0.0447	**0.2738**	-0.0802
MIS	p	0.47	0.94	0.97	0.27	**0.044**
	R	0.09	-0.01	0.004	0.14	**0.25**
N/L	p	**0.025**	**0.013**	**0.0048**	0.052	0.39
	R	**-0.28**	**-0.3**	**-0.34**	-0.24	-0.11

C-reactive protein; MIS malnutrition inflammation score, N/L neutrophils/lymphocytes ratio. r: correlation coefficient. *p < 0.05.

## Discussion

Various modifications of gMB composition have been described in CKD patients. The relationship between CKD and gMB is probably bidirectional, since kidney diseases may disrupt a balanced gMB and in turn alterations in gMB could affect kidney disease progression and the degree of the associated comorbidities (e.g. CV complications).

It is also acknowledged that frailty is very common in CKD patients, particularly in elderly subjects, with high impact on their quality of life and clinical outcomes. Frailty is also associated *per se* with changes in gMB composition [24,25].

In our study, the prevalence of frailty in the CKD cohort was 59%, which was consistent with data previously published [1,26]. F-CKD patients had higher MIS compared with NF-CKD, suggesting that frailty is associated with higher degree of inflammation and malnutrition. The main aim of this study was to evaluate whether there were differences in gMB composition between F and NF-CKD individuals. However, our results cannot clarify whether a direct cause-effect relationship does exist between gMB changes and frailty in CKD patients or both these findings are merely associated through other common causal factor(s) (e.g. inflammation).

Our results obtained with the PCR-DGGE method show that gMB profiles of C group cluster into two main groups, one of which was characterized by a trend toward a greater inter-individual variability, compared to CKD patients. The only published study which assessed gMB through PCR-DGGE method in 20 CKD non-dialysis patients, did not demonstrate any difference compared with 19 controls [27]. On the other hand, we did not find any significant difference in the richness of bands between the gMB profiles of CKD (F and NF) and controls.

However, the Illumina technique, though confirming no significant differences in α diversity of the compared groups, found some distinctive features in gMB composition in CKD compared with C and in F-CKD compared with NF-CKD. In particular, CKD patients showed a significantly increased abundance of bacteria (*Citrobacter*, *Anaerotruncus*, *Coprobacillus* genera and *Ruminococcus torques* species) which have been recently associated with other pathological conditions, such as IBD and vascular and inflammatory diseases [4, 17, 28–30]. *Citrobacter* and *Ruminococcus torques* are bacteria particularly prone to generate phenolic compounds, including p-cresyl sulphate (PCS) which accumulates in CKD patients, and has been suggested to play a causal role in cardiovascular complications [31,32]. Furthermore, *Citrobacter* species are also involved in the production of another uremic toxin, indoxyl-sulfate, through the conversion of tryptophan to indole. *Citrobacter* genus belongs to Enterobacteriaceae family, urease-producing bacteria whose abundance has been reported to be increased in CKD by other Authors [6,25,26]. However, we did not find any difference in the relative abundance of the other species belonging to the Enterobacteriaceae family between CKD patients and controls. This partial discrepancy could be explained by the fact that previously published studies analyzed gMB composition in ESRD patients (often undergoing dialysis treatment), whereas our cohort was composed of individuals affected by CKD 3b-4, which is characterized by a less severe degree of inflammation.

Consistently with existing literature, we found a reduced abundance of saccharolytic and butyrate- producing bacteria (*Prevotella*, *F*. *prausnitzii*, *Roseburia*) in CKD patients respect to controls. Butyrate seems to play an important role in the maintenance of gut barrier function: it promotes colon motility; reduces inflammation; increases visceral vascularization; inhibits tumor cell progression; induces differentiation of T-regulatory cells, etc. [33]

It is widely agreed that lactobacilli play a beneficial role in the intestinal tract. As a matter of fact, in the few data derived from animal studies and small CKD cohorts, a decreased abundance of lactobacilli was associated with reduced renal function [8, 11, 34]. Unlike previous studies, we found that the abundance of *Lactobacillus* genus was even higher in CKD patients compared with controls. A non-statistically significant trend towards higher lactobacilli counts in both hemodialysis and CKD patients, compared with healthy controls, was also reported in a previous study, which applied culture methodology [35]. We observed that *Lactobacillus*, *Coprobacillus* and *Anaerotruncus* genera have a higher abundance not only in CKD patients respect to controls, but also in F respect to NF patients. Though the two latter bacteria have been already associated with a worse state of health, there is not a counterintuitive explanation for the finding of increased *Lactobacillus* genus abundance in F-CKD patients, given that this bacterium is commonly considered as a beneficial one.

In an attempt to reconcile these discordant data, we should consider that observations from Vaziri *et al* [8] were based on experimental studies in rats, which are not completely comparable with the results obtained in humans. On the other hand, Wang *et al* study [11] was performed in a small cohort of peritoneal dialysis patients, showing a reduced abundance of only two lactobacilli species (*Lactobacillus plantarum and Lactobacillus paracasei)*.

Moreover, it should be considered that also lactobacilli, as some other genera (*Clostridium*, *Bacteroides*, *Enterobacter*, *Bifidobacterium)*, can metabolize aromatic amino acids to phenolic compounds (including PCS) which accumulate in CKD, since their clearance is mainly renal [36]. Therefore, some lactobacilli species might contribute to the production of toxic compounds, although few interventional studies showed that their administration as probiotics was followed by a reduction of PCS levels [37]. Alternatively, the increase in lactobacilli could simply be due to the reduction of competitive species and therefore being the consequence of an altered balance of the microbial environment, as described in other chronic diseases [38].

It is noteworthy that although no apparent differences in macronutrients and fiber intake were found between F-CKD, NF-CKD and Controls through dietary diary analyses, 24 patients in the CKD cohort (13 F-CKD and 11 NF-CKD) were prescribed aproteic food, that may have influenced this finding since it is enriched with fibers and inulin, potentially acting as prebiotics and supporting the growth of lactobacilli [39,40].

Regarding associations of gMB composition with clinical parameters and inflammatory indices, we found that a greater mean abundance of unclassified Mogibacteriaceae and *Oscillospira* genus was positively correlated with CRP and MIS levels, respectively, and that *Akkermansia*, *Ruminococcus and Eubacterium* genera, traditionally considered to be associated with a healthy gMB, were negatively related to N/L ratio.

Regarding the associations between gMB composition and frailty, we found a significant increase in the abundance of several bacterial species in F-CKD respect to NF-CKD. Many of these bacteria (Coriobacteriaceae family, *Anaerotruncus*, *Coprobacillus* and *Dorea* genera and *Eubacterium dolichum and Eggerthella lenta* species) have already been associated to frailty in various studies [6, 24, 25, 41, 42]. Some of them (*Eggerthella*, *Coprobacillus* and *Oscillospira* genera) have been previously described as correlates of biological aging or abundant in elderly individuals (Mogibacteriacaee, Coriobacteriaceae families and *Lactobacillus*, *Eubacterium cylindroides* species) [43,44]. Furthermore, some bacteria seem to be positively correlated also with different nutritional and physical features: *Eubacterium dolichum* has been associated with obesity and western diet in mice, and *Oscillospira* genus has been found in high concentrations in the stools of lean subjects [44,45]. The latter has been also associated with urinary PCS levels and positively correlated with age in previous studies [28,44]. In our cohort, *Oscillospira* genus significantly correlated with MIS, which is significantly higher in F-CKD individuals. Nevertheless, although F-CKD have lower lean mass compared with NF-CKD, this difference was not statistically significant. These results might suggest that frailty could be in part dependent more on functional than structural changes of muscular and/or neurological systems.

In conclusion, many alterations in gMB composition seem to be related to both CKD and frail condition. If this might play a casual role in frailty through an unbalanced production of toxic substance(s), or if gMB changes are merely a consequence of different dietary and life-style behaviors of frail patients, it cannot be explained by the present study and all the yet available data. Further studies, possibly utilizing new high-throughput tools, will be required to understand the potential correlations between gMB composition and other inflammation and oxidative stress markers in these patients. If a cause-effect relationship between gMB alterations and frailty in CKD will be demonstrated, the restoration of a healthy gMB, using pro- or prebiotics or through the correction of lifestyle and dietary behaviors, could be a strategy for reducing the comorbidity burden of CKD patients.

Our study has a design limitation regarding the small sample size of the control group that prevented us from performing a case-control analysis. This could have biased gMB differences that were found between CKD and healthy control groups. However, we believe that the major finding of our study is the significant difference of gMB composition found between F-CKD and NF-CKD patients.

## Supporting information

S1 Fig Distribution of bacterial families, expressed as relative abundance, in samples of control group (C), CKD frail (CKD-F) and not frail (CKD-NF) subjects. (TIF)

S2 Fig Bacterial species found to be statistically different between CKD- F, -NF and Controls at high-throughput sequencing are expressed below as relative abundance normalized with cumulative-sum scaling (CSS) at the ANOVA analysis. Blue, red and yellow bars represent Frail, Not frail and Controls respectively. *p < 0.05, **p < 0.01.
(TIF)

S1 Table (XLS)
