# Supplementary figures and images for: Gut microbiota composition and frailty in elderly patients with Chronic Kidney Disease
Source: PLoS One. 2020 Apr 1;15(4):e0228530. doi: 10.1371/journal.pone.0228530 (PMC7112193; doi:10.1371/journal.pone.0228530)

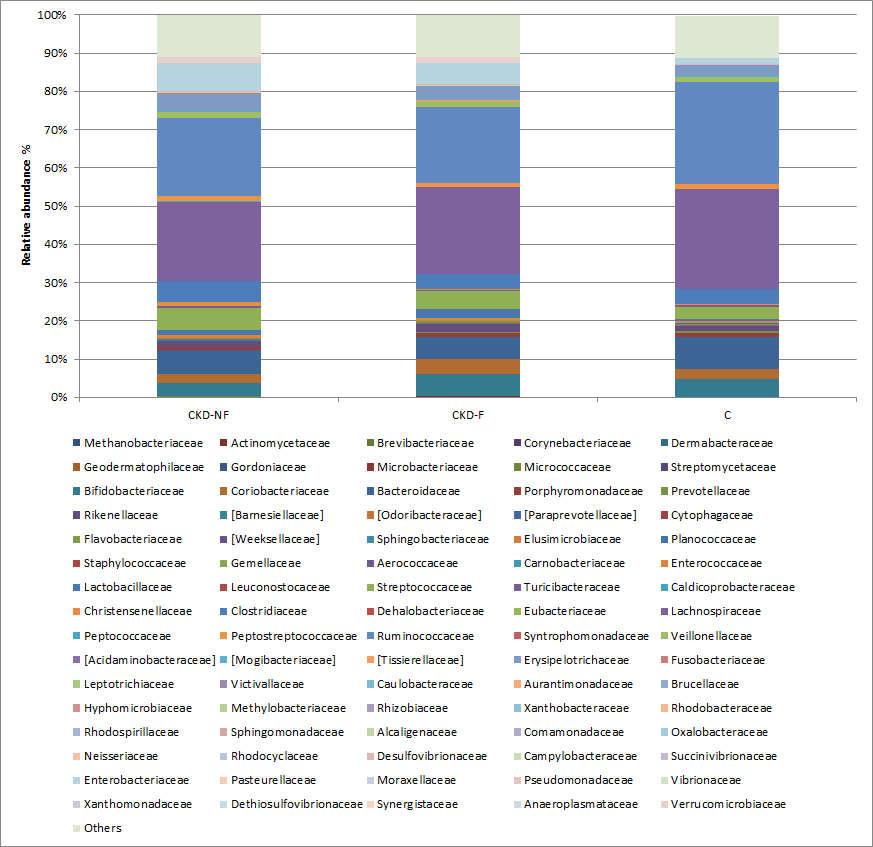

Supplement: S1 Fig — (TIF) [file pone.0228530.s001.tif]

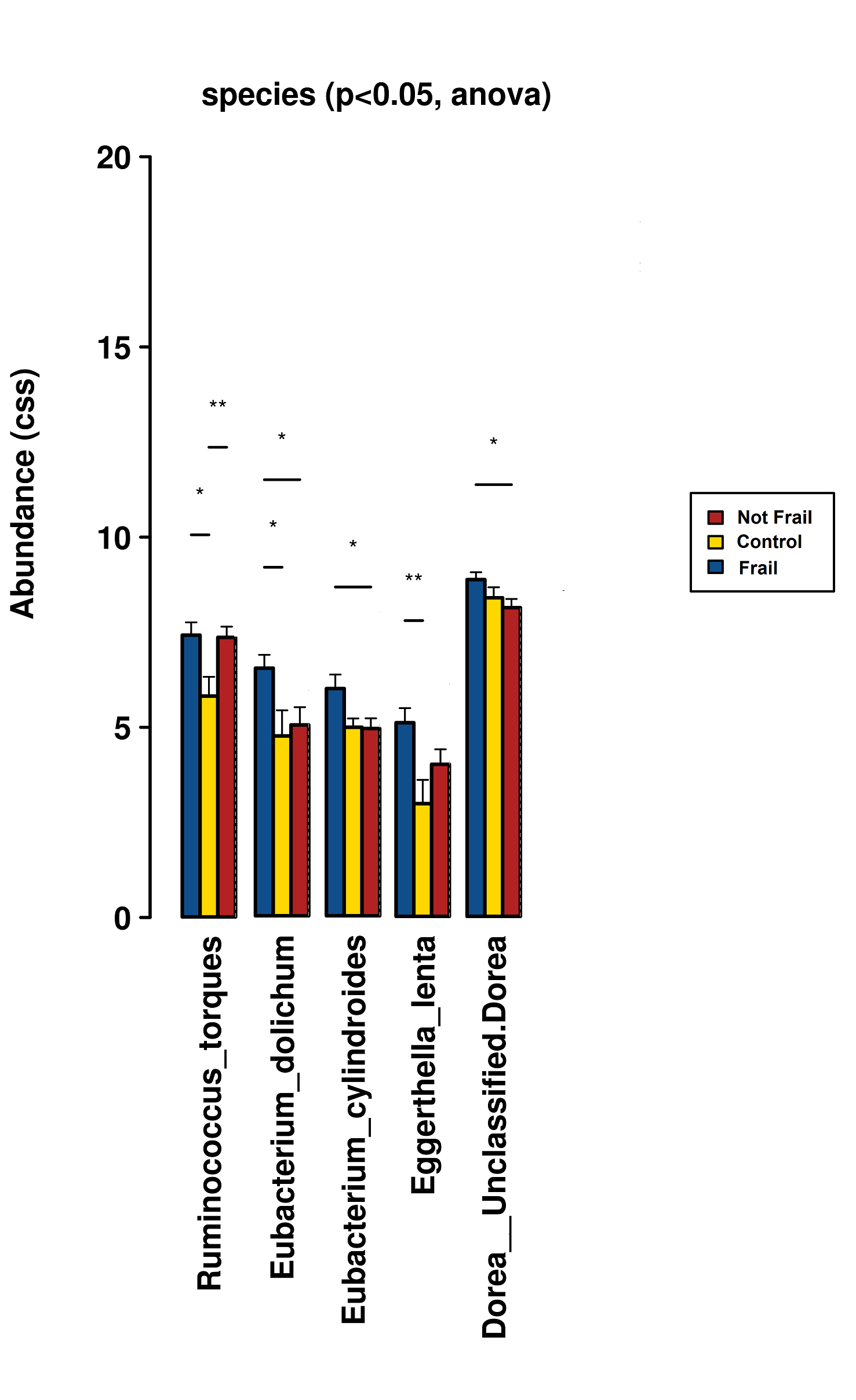

Supplement: S2 Fig — Blue, red and yellow bars represent Frail, Not frail and Controls respectively. *p < 0.05, **p < 0.01. (TIF) [file pone.0228530.s002.tif]
